# Why the Term MINOCA Does Not Provide Conceptual Clarity for Actionable Decision-Making in Patients with Myocardial Infarction with No Obstructive Coronary Artery Disease

**DOI:** 10.3390/jcm10204630

**Published:** 2021-10-09

**Authors:** Francesco Pelliccia, Mario Marzilli, William E. Boden, Paolo G. Camici

**Affiliations:** 1 Department of Cardiovascular Sciences, Sapienza University, 00166 Rome, Italy; 2 Department of Surgery, Medical, Molecular and Critical Area Pathology, University of Pisa, 56121 Pisa, Italy; mario.marzilli@med.unipi.it; 3 Department of Cardiology, VA New England Health Care System, Boston, MA 02101, USA; William.Boden@va.gov; 4 Department of Cardiology, San Raffaele Hospital and Vita e Salute University, 20100 Milan, Italy; camici.paolo@hsr.it

**Keywords:** MINOCA, myocardial infarction

## Abstract

When acute myocardial injury is found in a clinical setting suggestive of myocardial ischemia, the event is labeled as acute myocardial infarction (MI), and the absence of ≥50% coronary stenosis at angiography or greater leads to the working diagnosis of myocardial infarction with non-obstructed coronary arteries (MINOCA). Determining the mechanism of MINOCA and excluding other possible causes for cardiac troponin elevation has notable implications for tailoring secondary prevention measures aimed at improving the overall prognosis of acute MI. The aim of this review is to increase the awareness that establishing the underlying cause of a MINOCA is possible in the vast majority of cases, and that the proper classification of any MI should be pursued. The initial diagnosis of MINOCA can be confirmed or ruled out based on the results of subsequent investigations. Indeed, a comprehensive clinical evaluation at the time of presentation, followed by a dedicated diagnostic work-up, might lead to the identification of the pathophysiologic abnormality leading to MI in almost all cases initially labeled as MINOCA. When a specific cause of acute MI is identified, cardiologists are urged to transition from the “all-inclusive” term “MINOCA” to the proper classification of any MI, as evidence now exists that MINOCA does not provide conceptual clarity for actionable decision-making in MI with angiographically normal coronary arteries.

## 1. Introduction 

According to the traditional view, myocardial ischemic conditions are closely linked to coronary atherosclerosis, with the progressive narrowing of the vessel lumen by plaque growth being the background of chronic myocardial ischemia (“stable angina”), and thrombus superimposition on a ruptured or fissured or eroded plaque being the precipitating mechanism of acute myocardial ischemia [1]. This (mis)conception has dictated diagnostic, therapeutic, and prognostic approaches to cardiac ischemic conditions for decades [2]. However, in recent years, an overwhelming body of evidence has challenged this model and additional mechanisms of myocardial ischemia have been identified [3]. Scientific guidelines eventually acknowledged the multifactorial nature of “stable angina” and proposed a nomenclature shift for this condition in 2019, suggesting the term “chronic coronary syndromes” [4]. The implications of this new terminology, expressing a major change in the understanding of chronic myocardial ischemia, are deep and wide. It will take time to achieve a full application in clinical practice, but the cultural process has been activated [5]. Paradoxically, for acute ischemic conditions, the nomenclature has shifted from disease to syndrome; thus, admitting the multifactorial nature occurred long ago, but with limited if any effect in clinical practice. Indeed, diagnostic and therapeutic strategies are still focused on the identification and removal of atherosclerotic obstructions [6]. Whenever a patient presents with typical features of myocardial infarction (MI) but without coronary atherosclerotic obstructions, the cardiologist’s reaction is of surprise and disbelief, and the diagnosis is challenged or openly denied. Despite many reviews and position statements from both the European and American societies [7,8,9,10], too many clinicians still suppose that the absence of obstructive coronary artery disease (CAD) excludes the possibility of an acute MI [10].

## 2. Discussion

### 2.1. Myocardial Infarction with No Obstructive Coronary Artery Disease

The observation that myocardial infarction (MI) can occur in patients with angiographically normal coronary arteries was first made in the 1970s [11,12,13,14,15]. The first reports already noted that the latter category of patients tended to be different from the much larger group of patients who had evidence of myocardial necrosis associated with coronary atherosclerosis. Since the early case reports and subsequent small collected series, significant advances in our understanding of the pathophysiologic features of MI with normal coronaries (MINCA) have occurred. The term MINOCA was firstly introduced by John Beltrame in 2013. John Beltrame proposed to replace the former terminology (MINCA) that included only patients without atherosclerosis of the epicardial vessels with the term MINOCA in order to encompass patients with angiographic stenoses ranging between 1% and 50% [16,17]. This proposal, though conceptually important and useful to increase the awareness that ischemic myocardial necrosis can occur in patients without visible coronary atherosclerotic obstructions, has hindered with time our understanding of the pathophysiology of MI. Indeed, MINOCA has been progressively regarded as a separate entity, thus perpetuating the misconception that only infarctions in patients with obstructive CAD are “true infarctions”. Moreover, even cardiologists that accept the idea of an acute MI occurring in patients without lumen obstructions regard MINOCA as a more benign condition than classic MI-CAD. Notably, a large body of evidence suggests otherwise. In an autopsy study among young people with fatal ischemic heart disease, 38% of females and 23% of males were found to have no significant coronary artery disease [18]. In the VIRGO study, patients with MINOCA had similar mortality rates and comparable quality of life to patients with MI-CAD [19]. The Korean Infarct Registry showed that MINOCA patients had a similar risk of major adverse events as MI-CAD patients [20]. Moreover, approximately 25% of patients with MINOCA will experience angina in the subsequent 12 months, which is similar to the frequency reported in patients with MI-CAD [21].

**Figure 1:**
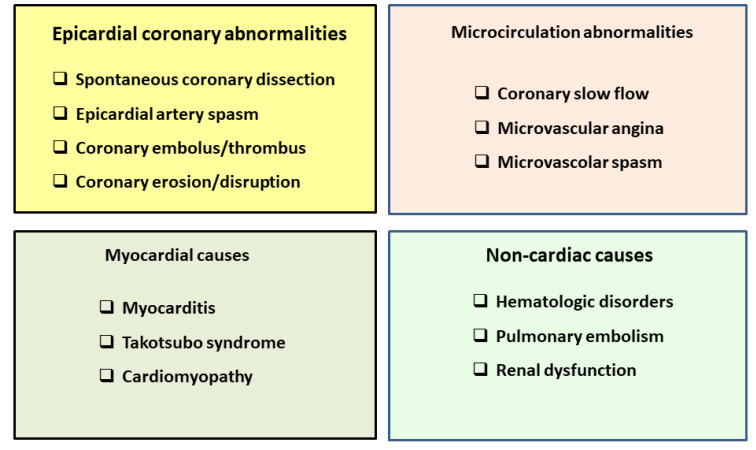
Pathophysiologic mechanisms of MINOCA.

The next challenge facing cardiologists after becoming aware of the prevalence and relevance of MINOCA will be the acknowledgment that MINOCA is not a different MI, but simply an MI with a different etiology. Several precipitating mechanisms have been proposed for MINOCA and include many of the mechanisms considered for chronic ischemic syndromes: epicardial coronary vasospasm, coronary microvascular dysfunction, coronary embolism/thrombosis, spontaneous coronary artery dissection, and non-vascular mechanisms (supply–demand mismatch) (Figure 1) [10]. Similarly to chronic ischemic syndromes, this list should not be considered exhaustive, but preliminary and incomplete. However, the need for a distinct name, MINOCA, expresses the “hard to die concept” that a severe stenosis is the “conditio sine qua non” for the development of “real” acute MI. This bias comes from the common (mis)belief that a linear relation exists between stenosis severity and coronary blood flow (CBF). The pioneering studies of Gould et al. in the 1970s elegantly demonstrated that resting CBF does not decrease until there is stenosis >85% [22]. This evidence was confirmed almost 20 years later. Myocardial blood flow (MBF) was measured non-invasively using positron emission tomography (PET) and ^15^O-labeled water, and stenosis severity was assessed by quantitative coronary angiography (QCA), in patients with single vessel coronary artery disease (CAD) [23]. The results of this study demonstrated that baseline MBF remained constant despite the increasing severity of the stenosis, even in patients with >80% luminal diameter reduction. In keeping with the observations of Gould et al. in the canine model [22], hyperemic MFB in patients was found to decrease progressively only from stenoses >50% and resting blood flow was not different from baseline MBF until stenosis severity was >80% [23].

More recent studies have once again confirmed the lack of a relationship between stenosis severity and myocardial perfusion. In a study in which stenosis hemodynamic severity was assessed by fractional flow reserve (FFR) and the cross-sectional vessel area was measured by intravascular ultrasounds, no linear relation was found between these two parameters, with normal FFRs measured even in vessel lumens as narrow as 1 square mm [5]. Moreover, in a study where myocardial perfusion was assessed by PET and stenosis severity was estimated by computed tomography angiography (CTA), several patients with complete coronary occlusions were found to have normal myocardial perfusion, and many patients with fully patent coronary vessels were found to have severe perfusion abnormalities [24].

A further step in the understanding of acute ischemic syndromes will be the acknowledgement that, similarly to what was admitted for chronic ischemic syndromes, different mechanisms may overlap in the same patients and that the precipitating mechanism(s) may change in time [3,4]. The clinical implications of this new understanding will include the awareness that, e.g., a vasospastic mechanism may be active not only in patients with “clean” coronary arteries, but also in patients with atherosclerotic lesions and vice versa. After all, it has long been known that thrombosis is a very dynamic phenomenon and the underlying atherosclerotic plaque can be non-obstructive [25]. The conclusive message is that MINOCA and MI-CAD may be associated with different precipitating mechanisms, but share identical pathologic characteristics, diagnostic criteria, and prognostic implications. In practice, detection of myocardial ischemic necrosis, the essence of MI, should be perceived as a first step, to be followed by the search for the precipitating mechanism(s). However, the term MINOCA is quite imprecise and amorphous and does not clearly imply that an MI can be secondary to several causes that can occur with highly variable degrees of coronary atherosclerosis, ranging from 0% to 50%. Most importantly, “MINOCA” can erroneously be regarded as a distinct entity, thus not impacting the diagnostic and therapeutic clarity. Conversely, the term “MINOCA” should merely describe a moment in the diagnostic pathway of the patient and arguably should not be considered a diagnosis. 

### 2.2. The Emerging Concept of MINOCA as a “Working Diagnosis”

The identification of the underlying cause of myocardial necrosis in the individual patient is crucial in choosing the optimal treatment and possibly in improving clinical outcomes. As recently proposed, MINOCA is a “‘working diagnosis’ that should prompt further investigations in order to ascertain the etiology in all cases” [9,26]. We are expanding this concept, suggesting that MI should be “only” considered a working diagnosis, prompting further investigations aiming at the identification of the responsible mechanisms, keeping in mind that the presence of an atherosclerotic plaque does not exclude the presence of other mechanisms such as vasospasm or microvascular dysfunction [27]. MINOCA encompasses a heterogenous group of conditions that include both atherosclerotic and non-atherosclerotic disease resulting in myocardial damage that is not due to obstructive coronary artery disease. In many ways, it is a term that describes a moment in the diagnostic pathway of the patient and is arguably not a diagnosis. Accordingly, the time has come for a paradigm shift, as evidence now exists that a comprehensive clinical evaluation at the time of presentation, followed by a dedicated diagnostic work-up, might help to identify the pathophysiologic mechanism that has caused an MI in almost all cases initially labeled as MINOCA. With this novel approach, a more sensible classification of acute MI can be reached without relying on the nonspecific term MINOCA. Irrespective of the presence or absence of obstructive CAD, MI is a pathologic nomenclature for ischemic myocardial cell death [28]. Consequently, fundamental to the diagnosis of acute MI is the demonstration of elevated cardiac biomarkers of necrosis, typically a cardiac troponin >99th percentile of the upper reference level. Rise and fall of troponin levels are consistently associated with myocyte injury [28]. Accordingly, the evidence of myocyte necrosis should prompt further investigations in order to ascertain its specific cause. Research has clearly highlighted that MINOCA can indeed be caused by a wide array of pathophysiologic mechanisms (Figure 1). These include epicardial coronary abnormalities, such as spontaneous coronary dissection, epicardial artery spasm, and coronary embolism. A further possible cause is disruption of an atherosclerotic plaque, although the causal link between coronary artery erosion and MI cannot always be demonstrated beyond doubt. It is worth noting that myocardial ischemia, albeit caused by different mechanisms, is at the core of MINOCA [29,30], as in the case of Takotsubo syndrome (TTS), which is currently included within the spectrum of acute coronary syndromes [31]. An accurate diagnosis has relevant therapeutic implications, and this observation might explain the reasons that pharmacologic agents that are class I options for MI-CAD have failed to show significant benefits in patients with a provisional diagnosis of MINOCA.

### 2.3. Back to the Future: Clinical Triage in Provisional MINOCA

At time of presentation, several clinical characteristics may suggest the mechanism precipitating MI. The clinical presentation (i.e., preceding events and demographics), electrocardiographic features (i.e., unusual patterns), and biochemical data (i.e., atypical elevations in troponin levels) may help to plan an expedited diagnostic work-up. For example, symptoms associated with coronary artery vasospasm typically occur at rest, often during sleep, or with minimal effort, such as walking in the early hours of the morning, and are associated with severe arrhythmias, ranging from heart block to ventricular tachycardia [32]. Meticulous history-taking to find evidence supporting characteristic circadian or diurnal variation of the angina threshold might allow the accurate diagnosis of abnormal epicardial vasomotion as a cause of acute MI. TTS occurs more often in elderly women who have often experienced severe, unexpected emotional or physical stress, and a have a former diagnosis of anxiety or panic disorder [33]. In these patients, pain extends from the chest to the neck and into the head, consistent with the acute catecholamine surge, and is frequently associated with heightened anxiety [34,35]. Finally, acute myocarditis should be suspected in young patients (<50 years) with no history of cardiac disease who have viral prodromes (i.e., fever, arthralgia, fatigue) or signs of connective tissue disease [36]. Additional findings may suggest an underlying disease in non-infectious etiologies. Moreover, patients with eosinophilic myocarditis will have a pruritic maculopapular rash. When the pericardium is involved (myo-pericarditis), a friction rub also may be heard on cardiac auscultation. The diagnosis of non-atherosclerotic MI is more common in the female gender, likely because the prevalence of coronary atherosclerosis is lower in women than men, though sex-related mechanisms might contribute [37].

### 2.4. The Crucial Role of a Complete Diagnostic Work-Up in MINOCA

**Figure 2:**
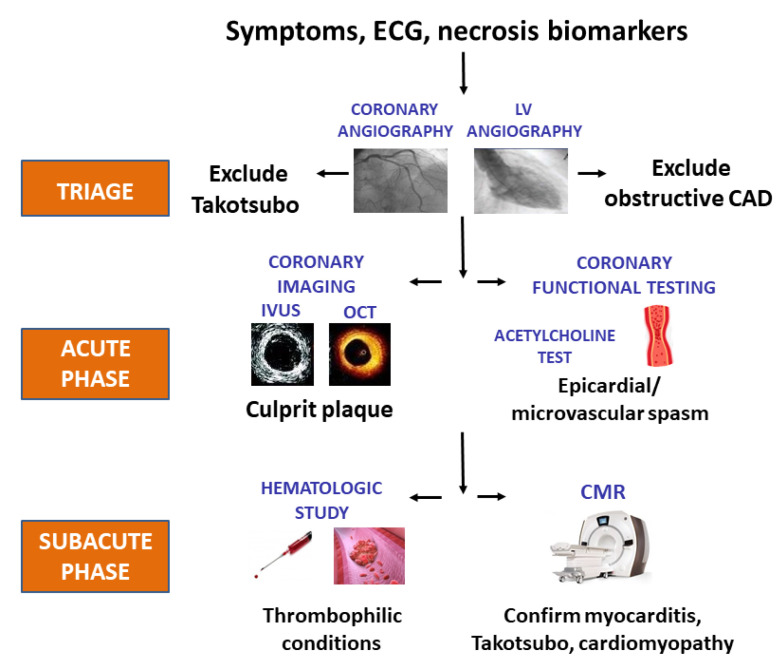
Step-by-step diagnostic work-up to unravel the causes of MI in MINOCA. ECG: Electrocardiography; CAD: coronary artery disease; LV: Left ventricle; OCT: optical coronary tomography; CMR: cardiovascular magnetic resonance; IVUS: intravascular ultrasound; MI: myocardial infarction; MINOCA: myocardial infarction with non-obstructed coronary arteries.

Thanks to invasive and non-invasive diagnostic tools, the precipitating mechanism of acute MI can be easily identified in most patients with and without atherothrombotic obstructions (Figure 2). In the acute phase of MINOCA, beyond coronary angiography, additional intracoronary imaging methods, such as intravascular ultrasound (IVUS) or optical coherence tomography (OCT), can identify alternative atherosclerotic mechanisms, such as plaque rupture, erosion or ulceration, or spontaneous coronary artery dissection [38]. Patients in whom intracoronary imaging does not provide any diagnostic information may undergo coronary provocative testing soon after the acute phase of MI, possibly in a staged procedure at the time of the index hospitalization. Coronary artery spasm causing obstruction of ≥1 epicardial vessels can cause an MI defined of unknown etiology if no provocative testing is performed. Vasoconstriction leading to MI can also occur distally, at the site of coronary microcirculation. Unfortunately, the burden of both epicardial and microcirculation coronary spasm is largely underestimated, as provocative tests eliciting coronary artery vasoconstriction in susceptible individuals are not used on a “routine basis” in patients with MINOCA, despite their safety being clearly demonstrated [39,40]. In the recovery phase of MI, the role of cardiac magnetic resonance (CMR) in identifying mechanisms for MINOCA is well established. Along with cardiac imaging, hematologic assessment should be always performed in any MI, as the detection of blood disorders may deeply influence the subsequent therapeutic strategy [41].

Available evidence clearly supports the ability of a complete diagnostic work-up in unraveling the pathophysiologic mechanisms of MINOCA. Recently, Pelliccia et al. reviewed data from 82 studies including 8457 patients with MINOCA [42]. In the acute phase, 16 studies with IVUS or OCT (1207 patients) disclosed that plaque disruption and spontaneous coronary artery dissection had a pooled prevalence of 38% and 16%, respectively. In 18 studies, coronary function testing (1449 patients) showed a pooled prevalence of spontaneous and/or provoked epicardial coronary spasm of ~28%. In three studies (456 patients), the pooled prevalence of CMD was ~32%. In the subacute phase, 42 CMR studies (5821 patients) showed a pooled prevalence of myocarditis, TTS, and cardiomyopathy of 26%, 11%, and 7%, respectively. In 12 studies on thrombophilia screening (*n* = 834), the pooled prevalence of thrombotic disorder was ~11%. On the basis of these results, Pelliccia et al. concluded that the pathophysiology of MINOCA can be established in the majority of cases to provide direction for appropriate management [42].

Unfortunately, the lack of a clear sequence in the use of invasive and non-invasive tools in currently available algorithms for MINOCA challenges their implementation [43,44]. A recent study has highlighted the importance of multimodality imaging based on the use of coronary evaluation (i.e., with OCT) during the acute diagnostic coronary angiogram followed by cardiac imaging (i.e., with CMR) performed with a median of 6 days of infarct onset [45]. This multicenter study identified the cause for the suspected MINOCA patients in 85% of patients (64% MI, 15% myocarditis, 3% TTS, and 3% cardiomyopathy), which was better than either of the diagnostic technologies alone (i.e., 44% for OCT and 74% for CMR imaging alone), and reflects the ability to correlate the findings of the two diagnostic technologies. Hence, multimodal coronary imaging to identify plaque disruption and/or thrombosis that may be responsible for the MI, in conjunction with structural imaging of the myocardium for ischemic and non-ischemic causes that might account for the clinical presentation, will represent future optimal care [45].

## 3. Conclusions

With recent advances in diagnostics, the time is ripe for introducing a new paradigm in the assessment of patients with a provisional diagnosis of MINOCA. The pursuit of a proper classification of patients with MI on the basis of the underlying etiology cannot be postponed any longer, as a correct diagnosis has dramatic therapeutic and prognostic implications. Identification of the mechanism causing acute myocardial ischemia should lead to a more accurate labeling of any single case of MI. At present, this approach has been proposed only in the case of TTS. In line with the recently released 4th Universal Definition of Myocardial Infarction [28], TTS is currently considered an entity with its own characteristics and prognosis, which is apart from the causes of “myocardial injury” or “infarction” and should not be included within the spectrum of MINOCA. 

A similar approach should be followed for all the other etiologies leading to MI. The term “MINOCA” could still be used only to indicate the need for a “working diagnosis” in those cases where the cause of their ischemic infarct (e.g., epicardial coronary artery spasm, microvascular dysfunction, or coronary emboli) needs to be elucidated so that appropriately targeted therapy can be initiated [26]. When a specific cause of acute MI is identified, cardiologists are urged to transition from the “all-inclusive” term “MINOCA” to the proper classification of MI, as evidence now exists that MINOCA does not provide conceptual clarity for actionable decision-making.
